# Chromosome level genome assembly and full-length transcriptome of blacktip trevally (*Caranx heberi*)

**DOI:** 10.1038/s41597-026-07782-3

**Published:** 2026-08-24

**Authors:** Mudagandur S. Shekhar, Vinaya Kumar Katneni, Sudheesh K. Prabhudas, Ashok Kumar Jangam, Ashok Selvaraj, Irine Maria Jose, Karthic Krishnan, Raymond Jesudhas Angel Jani

**Affiliations:** ICAR-Central Institute of Brackishwater Aquaculture, 75, Santhome High Road, MRC Nagar, Chennai, 600028 Tamil Nadu India

## Abstract

*Caranx heberi* (Bennett, 1830) commonly known as the blacktip trevally belongs to the family Carangidae and is a potential brackishwater aquaculture species. However, the limited genomic resources are hindering the efforts to study its genetic traits and their molecular basis. To bridge this gap, we generated a high-quality reference genome employing multiple sequencing strategies including PacBio Hifi reads (135x), Illumina short reads (150x), and Hi-C chromosome conformation capturing (180x). The high-quality genome assembly consisted of 159 scaffolds summing to 618.71 Mb and an N50 value of 26.72 Mb. Among these, 24 chromosome level scaffolds covered 97.5% of the total assembly. The genome contained 20.94% of repeat elements and 30,354 protein encoding genes. In addition, full-length transcriptomes were generated using the PacBio IsoSeq approach from seven tissues (gill, kidney, liver, muscle, heart, spleen, and intestine). The comprehensive genomic and transcriptomic resources developed in this study will facilitate the domestication and aquaculture development of *C. heber*i, as well as support research on its nutritional potential, ecological adaptations, and evolutionary biology.

## Background and Summary

The blacktip trevally, *Caranx heberi* (Bennett, 1830) is a large marine pelagic fish belonging to the Carangidae family, which includes jacks, trevallies, and horse mackerels. It is found to prefer moderately deep water over rocky and coral reefs as well as open sandy areas. *C. heberi* is distributed throughout the tropical and subtropical waters of the Indian and West Pacific Oceans^1^. In spite of its wide distribution and commercial value very little information is available on the general biology of *C. heberi* and genomics of the species is very poorly studied. This underscores the necessity for deciphering whole genome sequence of this fish that would encapsulate a broader spectrum of genetic information at the genome level. *C. heberi* is known for its fast growth rate^2^, high market value and, excellent meat quality due to high protein content and essential fatty acids^3^. Deciphering the whole genome of *C. heberi* can further give insights into the captive maturation and spawning of this species, which will help establish the culture of the fish in the future. To our knowledge, there is limited published reports on whole genome assembly of fish species belonging to family Carangidae. The draft genome assembly available for giant travelly, *Caranx ignobilis*, is estimated to be of 625.92 Mb with a contig NG50 length of 7.3 Mb and a scaffold NG50 of 46.3 Mb and 90% of the genome was contained in 25 of the 203 scaffolds^4^. The genome assembly of bluefin trevally (*Caranx melampygus*) resulted in 711 Mb genome with a contig NG50 of 1.2 Mb and the assembly was annotated with 33.1 K protein-coding genes^5^. The current genome assembly methods which couple PacBio reads and Hi-C data has made it easier to assemble chromosome level genomes as evidenced by the recent sequencing of many Brackishwater species^6–10^.

This study utilized 135x coverage (83.52 Gb) of PacBio Hifi DNA sequence data to generate a contig assembly of 618.76 Mb length in 169 contigs with N50 value of 19.81 Mb. The contigs were scaffolded with 180 x coverage (111.59 Gb) of Hi-C linked reads to generate a final assembly of 618.71 Mb length in 159 scaffolds with N50 value of 26.72 Mb and a mitochondrial genome of 16,591 bp. The assembly consisted of 24 chromosome scale scaffolds similar to the chromosome numbers reported for *Caranx praeustus, Caranx latus, Caranx lugubris, Caranx ignobilis*, and *Caranx sexfasciatus*^11–16^. The 24 chromosome scale scaffolds represented 97.5% of the overall assembly length. The RNASeq data and IsoSeq data capturing the transcriptomes of seven different tissues of the same specimen were also generated and were used for prediction of 30,354 protein encoding genes. The knowledge of an annotated *de novo* genome sequence of *C. heberi* with total number of genes coupled with RNAseq data that is made available in this study, would facilitate to establish the genes and other regulatory mechanisms controlling desirable traits in this species. This might potentially pave ways to expand the genomic resources and devising efficient management and future research for aquaculture development of carangoid fishes.

## Methods

### Specimen collection and identification

**Fig. 1:**
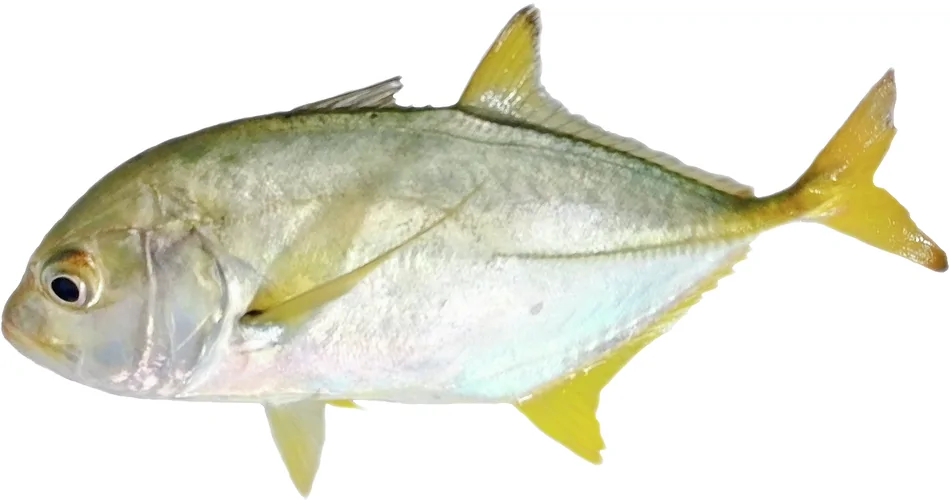
The specimen of *C. heberi* used for collecting the tissue samples for genome sequencing.

**Fig. 2:**
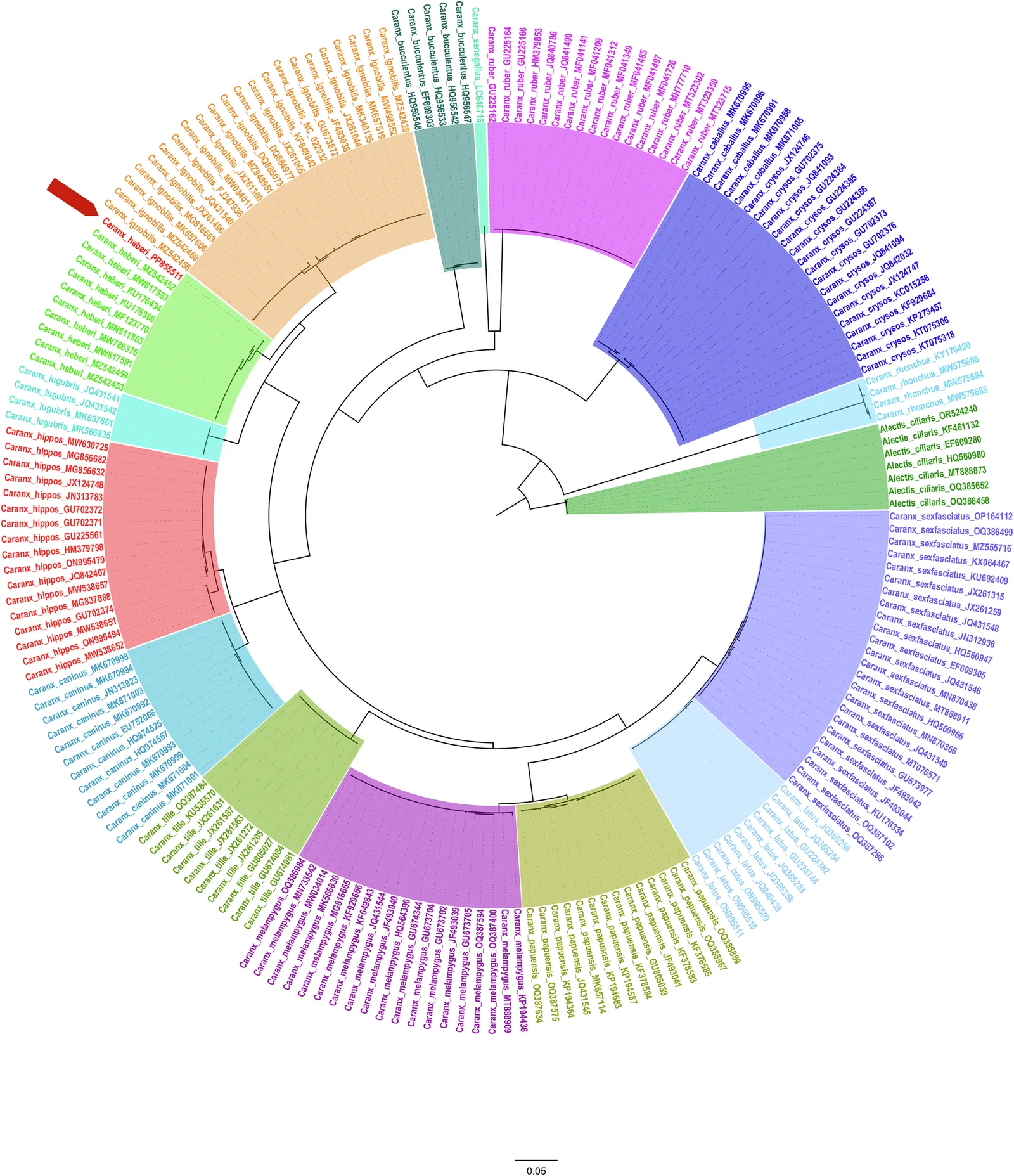
Maximum likelihood phylogenetic tree based on COI sequence of *Caranx* species and *Alectis ciliaris* as an outgroup. The specimen used in the current study was placed with with *C. heberi* clade as highlighted.

A single specimen of *C. heberi* was collected from the stock maintained at the Muttukadu Experimental Station of ICAR-Central Institute of Brackishwater Aquaculture, Chennai, India. The specimen was transported in sea water containers with aeration to maintain dissolved oxygen levels. The fish was anesthetized followed by euthanasia in accordance with CCSEA guidelines. The tissue samples were then flash frozen in Liquid nitrogen and stored at -80 °C and used later for genome and transcriptome data generation **(**Fig. 1**)**. The species identity was carried out sequencing of the Cytochrome C Oxidase I (COI) gene using primers F2 (5’ TCGACTAATCACAAAGACATCGGCAC 3’) and R1 (5’ TAGACTTCTGGGTGGCCAAAGAATCA 3’)^17^. The COI gene sequence was submitted to GenBank under the accession number PP855511. The phylogenetic tree based on the COI gene sequences available in the BOLD database for the genus *Caranx* (Table 1) was performed along with the COI sequence of *Alectis ciliaris* as an outgroup. The sequences were subjected to multiple sequence alignment with MUSCLE, and the overhanging ends were trimmed. The Hasegawa-Kishino-Yano model with Gamma and invariant site distribution rates (HKY + G + I), 1000 bootstrap iterations were used for the construction of a Maximum Likelihood tree using MEGA X software^18,19^. FigTree v1.4.32^20^ was used to visualize the phylogenetic tree (Fig. 2).

**Table 1:** List of accessions used for the construction of Maximum Likelihood phylogenetic tree.

Species Name	No. of Accessions	Accession Numbers
*Alectis ciliaris*	7	EF609280, HQ560980, KF461132, MT888873, OQ385652, OQ386458, OR524240
*Caranx bucculentus*	5	EF609303, HQ956533, HQ956542, HQ956547, HQ956548
*Caranx caballus*	5	MK670988, MK670991, MK670995, MK670996, MK671005
*Caranx caninus*	12	EU752066, HQ974525, HQ974567, JN313923, MK670992, MK670993, MK670994, MK670998, MK670999, MK671001, MK671003, MK671004
*Caranx crysos*	17	GU224384, GU224385, GU224386, GU224387, GU702373, GU702375, GU702376, JQ841093, JQ841094, JQ842032, JX124746, JX124747, KC015256, KF929684, KP273457, KT075306, KT075318
*Caranx heberi*	11	KU176366, KU176434, MF123770, MN511863, MW788376, MW817583, MW817591, MZ542452, MZ542453, MZ542459, PP855511*
*Caranx hippos*	17	GU225561, GU702371, GU702372, GU702374, HM379798, JN313783, JQ842407, JX124748, MG837888, MG856632, MG856682, MW538651, MW538652, MW538657, MW630725, ON995479, ON995494
*Caranx ignobilis*	22	DQ884977, DQ885073, FJ347936, GU673872, JF493038, JQ431540, JX261044, JX261360, JX261065, JX261496, KF649842, MG816663, MK348135, MK657519, MK657606, MW034011, MW498552, MZ542428, MZ542456, MZ542460, MZ948951, NC22932
*Caranx latus*	10	GU224382, GU224744, JQ365253, JQ365254, JQ365256, JQ365258, JQ840438, ON995509, ON995510, ON995511
*Caranx lugubris*	4	JQ431541, JQ431542, MK566835, MK657661
*Caranx melampygus*	19	GU673702, GU673704, GU673705, GU674344, HQ564390, JF493039, JF493040, JQ431544, KF649843, KF929686, KP194436, MG816665, MK566836, MN733542, MT888909, MW034014, OQ386984, OQ387400, OQ387594
*Caranx papuensis*	14	GU805039, JF493041, JQ431545, KF378583, KF378584, KF378585, KP194364, KP194587, KP194683, MK657114, OQ385889, OQ385967, OQ387575, OQ387634
*Caranx rhonchus*	4	GU805039, JF493041, JQ431545, KF378583
*Caranx ruber*	17	KF378584, KF378585, KP194364, KP194587, KP194683, MK657114, OQ385889, OQ385967, OQ387575, OQ387634, MF041485, MF041497, MF041726, MH777710, MT323302, MT323350, MT323715
*Caranx senegallus*	1	LC646716
*Caranx sexfasciatus*	24	EF609305, GU673977, HQ560947, HQ560966, JF493042, JF493044, JN312936, JQ431546, JQ431548, JQ431549, JX261259, JX261315, KU176334, KU692409, KX064467, MN870366, MN870438, MT076571, MT888911, MZ555716, OP164112, OQ386499, OQ387102, OQ387298
*Caranx tille*	10	GU674081, GU674084, GU805027, JX261205, JX261272, JX261563, JX261587, JX261631, KU535570, OQ387484

*Denotes the accession used in the current study.

## Sequence data generation

### PacBio Long read Hifi Sequence

Three individual muscle tissue samples were processed separately to extract high molecular weight genomic DNA using Blood and Cell Culture Midi Kit (Qiagen, Hilden, Germany). The quantity was estimated with a Qubit 4.0 fluorometer using the DNA HS assay kit (Thermofisher Scientific, USA). DNA purity was assessed using a NanoDrop 2000 spectrophotometer (Thermofisher Scientific, USA) and integrity was confirmed via 1% agarose gel electrophoresis. Approximately 67.5 µg, 53.8 µg, and 61.8 µg of total DNA were obtained from the three muscle tissue samples respectively. The Megaruptor 3 system (Diagenode, Belgium) was used to shear the DNA into approximately 20 kb fragments for preparation of SMRTbell libraries. Three separate libraries were prepared using the SMRTbell Express Template Preparation Kit 3.0 (Pacific Biosciences, California, USA). The library sizes were estimated using the Agilent FEMTO pulse analyser (Agilent Technologies, USA). The libraries with average size of 11.077 Kb, 14.009 Kb, and 15.826 Kb were subjected to purification with AMPure PB beads (Pacific Biosciences, USA) to remove fragments smaller than 5 kb. The purified size-selected libraries were subjected to primer annealing, polymerase binding using the Sequel II Binding Kit 3.2 (Pacific Biosciences, California, USA), and loaded onto three separate SMRT cells containing 8 M zero-mode waveguides (ZMWs) for sequencing on the PacBio Sequel II system in CCS/HiFi mode (Nucleome Informatics Pvt Ltd, India). The three libraries yielded 505 Gb, 452 Gb, 550 Gb of polymerase read bases, with a distinct molecular yield accounting for 95 Gb, 91 Gb, 100 Gb (Table 2) respectively. Raw polymerase reads were processed using the CCS v6.4.0 tool of the PacBio suite (–min-passes = 3;–min-snr = 2.5;–min-rq = 0.99) to generate HiFi reads.

**Table 2:** Summary of the PacBio sequencing data generated from *C. heberi* from 3 SMRT cells.

	Library 1	Library 2	Library 3
**Polymerase Read Bases**	550,570,591,859	452,767,669,752	550,228,941,473
**Polymerase Reads**	6,062,567	4,993,865	5,973,957
**Polymerase Read Length (Mean)**	90,814	90,664	92,104
**Polymerase Read N50**	198,327	213,633	206,528
**Unique Molecular Yield**	95,780,290,560	91,687,034,880	100,880,867,328
**Hifi Reads**	2,347,378	1,653,888	2,142,445
**Total Hifi bases**	26,313,223,978	26,361,380,217	30,844,192,727
**HiFi Sequence coverage (x)**	42.52	42.6	49.85
**Hifi reads-N50, (bp)**	16,355	20,837	17,610
**Longest Hifi read length, (bp)**	49,906	50,962	50,007

### Short read DNA sequence

Genomic DNA from the muscle tissue of *C. heberi* was extracted using the DNeasy Blood and Tissue Kit (Qiagen, CA). The quality and quantity of the extracted DNA was measured using both the Qubit 3 Fluorometer (Thermofisher Scientific, Massachusetts, USA) and Nanodrop 2000 (Thermofisher Scientific, Massachusetts, USA). The integrity of the DNA was assessed on a 1% agarose gel. A library with average insert size of 327 bp was prepared using Kapa Hyper plus kit (Roche, Basel, Switzerland) and sequenced on Novaseq 6000 platform (Illumina USA) using 2 × 150 bp paired-end chemistry. About 585 million reads were generated generating approximately 93.03 Gb (150x coverage) of data with 90.59% bases in Q30 **(**Table 3**)**. The short read DNA sequence data was used for genome size estimation.

**Table 3:** Summary metrics of the Illumina short read sequence and Hi-C sequence data.

	Illumina Short reads	Hi-C		
	Library 1	Library 1	Library 2	Library 3
**Raw reads**	585,100,742	326,516,342	103,479,292	307,776,770
**Total bases**	93,031,017,978	48,977,451,300	16,453,207,428	46,166,515,500
**Sequence Coverage x**	150.36	79.16	26.59	74.61
**Q20**	96.11%	97.34%	88.39%	97.26%
**Q30**	90.59%	92.92%	81.00%	90.87%
**GC content**	44.46%	44.28%	55.33%	46.90%
**Mean Read length (bp)**	159	150	159	150

### Hi-C reads

Three Hi-C cross linking libraries were prepared from the muscle tissues of a single specimen of *C. heberi*, the first Hi-C library preparation and amplification was carried out using the Proximo Hi-C Kit, animal (Phase Genomics, Washington, USA) as per the manufacturer’s instructions. The library quality was checked on Bioanalyzer 2100 (Agilent Technologies, California, USA) and then loaded onto an S4 flow cell at approximately 10 nM concentration and sequenced by the Novaseq 6000 system (Illumina, USA) using 2 × 150 bp paired-end chemistry. This sequencing resulted in generating approximately 48.98 Gb (79x coverage) of data from 326.5 million reads **(**Table 3).

The second and third Hi-C libraries were prepared using Epitect Hi-C kit (Qiagen, Hilden, Germany) as per manufacturer’s instructions. The quantity of the libraries was assessed using the Qubit 4.0 fluorometer (Thermo Fisher Scientific, Massachusetts, USA) using the DNA HS assay kit (Thermo Fisher Scientific, Massachusetts, USA). The library sizes were estimated on Agilent Tapestation 4150 (Agilent Technologies, California, USA) utilizing high sensitive D1000 screentapes (Agilent Technologies, California, USA). The libraries were sequenced on the Novaseq 6000 platform (Illumina Inc., California, USA) using 2x150bp paired-end chemistry. The libraries generated 16.45 Gb (26.59x coverage) and 46.17 Gb (74.62x coverage) from, 103.48 and 307.77 million reads respectively **(**Table 3**)**. The Hi-C reads were pre-processed with the fastp v0.12.4^21^ tool to remove adaptors and perform quality trimming.

### RNASeq data generation

A total of seven tissues (muscle, spleen, heart, gills, kidney, liver, and intestine) were collected from *C. heberi* fish and flash frozen in liquid nitrogen followed by storage at −80 °C. The total RNA extraction was carried out using TRIzol (DSS Takara, CA, USA) method and purification was done by using Nucleospin MN RNA cleanup kit (Machery Nagel, Germany). The extracted RNA was quantified using the RNA HS kit (Thermofisher Scientific, Massachusetts, USA) on the Qubit 3.0 fluorometer (Thermo Fisher Scientific, Massachusetts, USA) and Nanodrop 2000 (Thermofisher Scientific, Massachusetts, USA). The RNA integrity was evaluated using Bioanalyzer 2100 (Agilent Technologies, California, USA). The cDNA synthesis and RNA sequencing library preparation was carried out using KAPA HyperPrep kit (Roche, Basel, Switzerland). The library fragment analysis and insert size estimation was carried out using Bioanalyzer 2100 (Agilent Technologies, California, USA) following manufacturer’s protocol to evaluate the average insert sizes obtained for muscle (514 bp), spleen (464 bp), heart (447 bp), gills (464 bp), kidney (441 bp), liver (474 bp), intestine (442 bp). Sequencing was performed on Illumina NovaSeq 6000 using 2 x 150 bp paired end chemistry. The raw data statistics are tabulated in Table 4.

**Table 4:** RNASeq data generated from different tissues of *C. heberi*.

Tissue	Raw Reads	Total bases (Gb)	High Quality Trimmed reads	Q20%	Q30%	GC %
Muscle	73,460,308	11.45	69,308,968	97.26	92.96	48.76
Kidney	61,776,762	8.37	51,233,146	98.08	94.92	50.26
Intestine	64,607,050	9.58	60,260,758	98.21	94.91	50.21
Liver	47,920,164	6.44	39,809,298	98.42	95.43	51.60
Gills	65,119,982	9.99	61,096,636	97.60	93.59	47.42
Heart	83,084,818	12.44	76,419,668	97.64	93.65	47.48
Spleen	76,817,290	11.46	69,445,084	97.53	93.46	47.04

### IsoSeq data generation

To generate the long-read IsoSeq data, total RNA was isolated from seven tissues of *C. heberi* (muscle, spleen, heart, gills, kidney, liver, intestine) for transcriptome analysis and used for full length cDNA synthesis using NEBNext Single Cell/Low Input cDNA Synthesis & Amplification Module (New England Biolabs Inc., Massachusetts, USA). The full-length cDNA was quantified on Qubit 3.0 fluorometer (Thermo Fisher Scientific, Massachusetts, USA) using dsDNA HS kit (Thermo Fisher Scientific, Massachusetts, USA) and examined the amplified cDNA on Bioanalyzer 2100 (Agilent Technologies, California, USA) using High Sensitivity DNA Kit (Agilent Technologies, California, USA). The library was constructed using the SMRTbell Express template Preparation Kit 2.0 (Pacific Biosciences, California, USA) as per the protocol of the manufacturers. The library size was assessed using a Femto pulse system (Agilent Technologies, California, USA) revealing the average library sizes for different tissues, muscle (3,026 bp), spleen (3,813 bp), heart (3,298 bp), gills (3,241 bp), kidney (2,702 bp), liver (3,841 bp), intestine (3,254 bp). This was followed by primer annealing, and polymerase binding using Sequel II binding kit 3.1 (Pacific Biosciences, California, USA) to prepare the bound complex. About 80 pM of the libraries was loaded on SMRTcell 8 M and sequenced in PacBio Sequel II system at Nucleome Informatics Pvt. Ltd, Hyderabad, India. The raw subreads were processed as described in Katneni *et al*.^22^, initially the hifi reads were extracted from the subreads using *ccs* tool of the PacBio suite (https://github.com/PacificBiosciences/pbbioconda) and subjected to demultiplexing and primer removal using the *lima* tool of the PacBio suite (https://github.com/PacificBiosciences/pbbioconda). The *refine* and *cluster* modules of the *isoseq* tool of the PacBio suite (https://github.com/PacificBiosciences/pbbioconda) was utilized to generate the full-length non-chimeric (FLNC) reads and full-length transcripts respectively (Table 5).

**Table 5:** IsoSeq data generated from different tissues of *C. heberi*.

Tissue	Number of Polymerase Reads	Number of Polymerase bases (bp)	Number of FLNC reads	FLNC total length (bp)	Isoform level Full-length Transcripts	Total bases (bp)	Mean transcript length (bp)	Max transcript length (bp)	N50 (bp)	GC %
Muscle	649,582	36,727,668,004	468,582	1,042,041,075	22,874	55,707,278	2,435	8,980	2,688	48.10
Kidney	504,356	28,510,705,897	387,183	779,921,520	26,104	54,761,405	2,098	7,142	2,303	47.82
Intestine	540,703	30,387,543,848	420,426	851,078,561	14,176	31,121,032	2,195	8,029	2,446	48.36
Liver	600,829	34,942,480,353	452,971	1,224,964,011	31,630	88,916,920	2,811	11,038	3,131	47.58
Gills	430,090	24,793,467,856	328,882	825,359,826	26,193	67,949,010	2,594	9,445	2,820	47.25
Heart	477,803	27,517,779,835	361,516	869,892,128	27,431	69,986,367	2,551	9,334	2,793	47.24
Spleen	310,114	17,855,916,576	235,873	549,843,957	19,358	48,116,298	2,486	9,015	2,753	47.56

### Genome size estimation

**Fig. 3:**
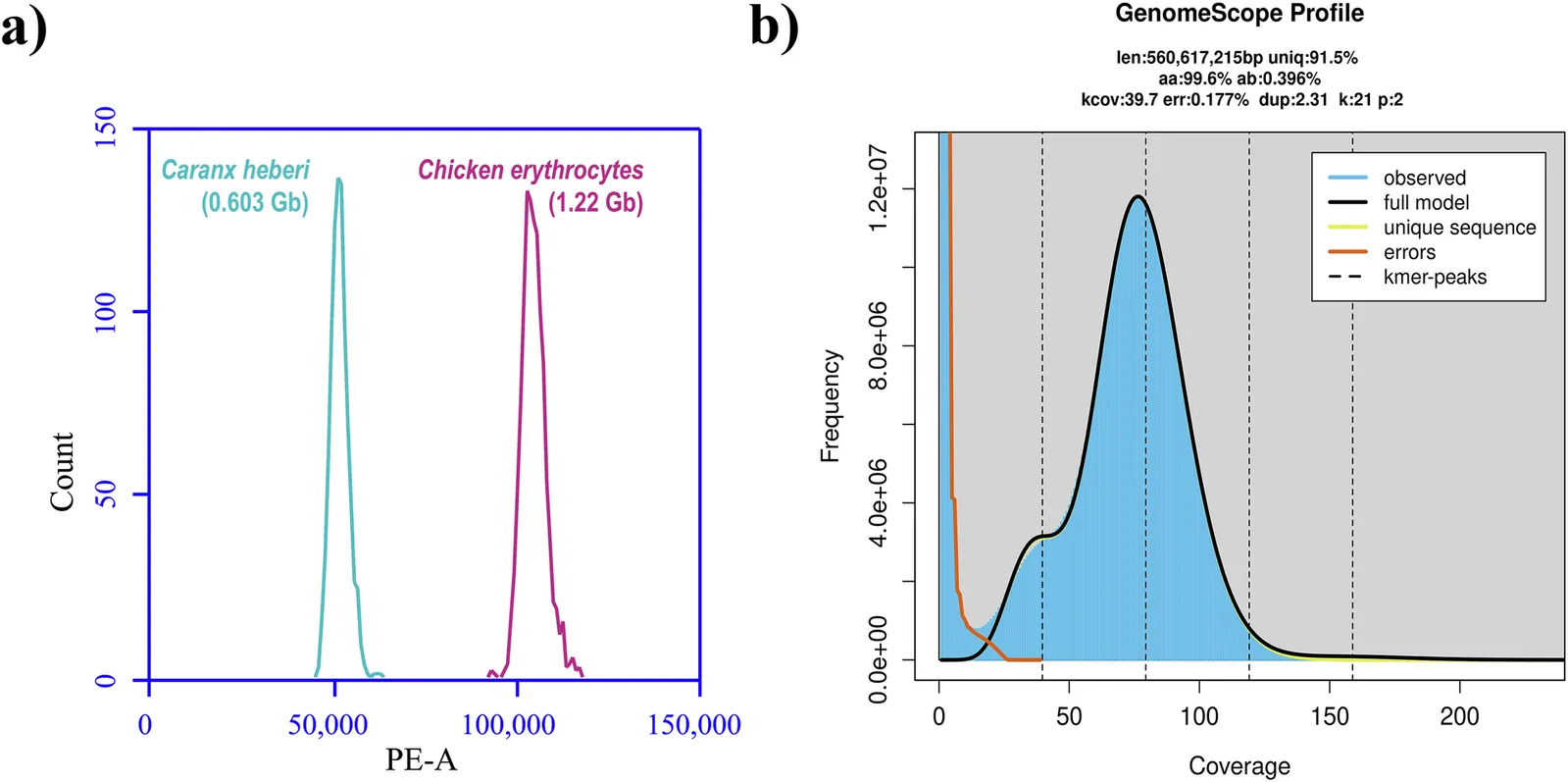
The genome size estimation of *C. heberi* (**a**) Flowcytometry graph depicting the fluorescent peaks of *C. heberi* and Chicken erythrocytes (**b**) K-mer based analysis profile using Jellyfish and Genomescope.

Flow cytometry-based genome size estimation was performed as mentioned^23,24^. Fish blood was collected in an anticoagulant solution (38 mM Citric acid, 76 mM Sodium citrate, and 122 mM D-glucose). The blood was centrifuged at 300 × g for 5 minutes at 4 °C, and the pellet was resuspended in 200 µl of 70% ethanol, followed by incubation on ice for 30 min. After centrifugation at 300 × g for 5 minutes at 4 °C, the pellet was then resuspended in 400 µl of phosphate-buffered saline. The resuspended cells (150 µl) were mixed with 600 µl of LB01 buffer (15 mM Tris, 2 mM disodium-EDTA, 0.5 mM spermine tetrahydrochloride, 80 mM KCl, 20 mM NaCl, and 0.1% v/v TritonX-100). The homogenized samples were filtered through a 40 µm sterile cell strainer (Corning Inc., USA). Approximately 500 µl of the filtrate was treated with 1 µl of RNase A (Qiagen, Germany), and nucleic acid content was stained with 12 µl of Propidium iodide (ThermoFisher Scientific, USA). Chicken erythrocytes (20 µl) from the BD^TM^ DNA QC Particles kit (BD Biosciences, USA) was mixed with LB01 buffer (480 µl), RNase A (1 µl), and propidium iodide (12 µl) for use as control. The tubes were incubated in the dark at 4 °C for 30 minutes before flow cytometry analysis. Flow cytometry readings were acquired on a BD Accuri^TM^ C6 flow cytometer (BD Biosciences, USA) for a minimum of 10,000 events using a slow flow rate (14 µl/min) and a core size setting of 10 µm. The histogram data was acquired on BD Accuri^TM^ C6 Plus software v1.0.23.1 (BD Biosciences, USA) for genome size estimation. The genome size of *C. heberi* was estimated to be 603 Mb based on the flow cytometry (Fig. 3a).

Genome size estimation of *C. heberi* was also performed based on short Illumina sequencing reads. Canonical 21 k-mers were counted from the illumina paired end reads using the program Jellyfish 2.2.3^25^ and the counts histograms were visualized using Genomescope^26^.The analyses has revealed the genome size of *C. heberi* to be 560.5 Mb with a heterozygosity of 0.396%. (Fig. 3b).

### Genome assembly of *C. heberi*

**Fig. 4:**
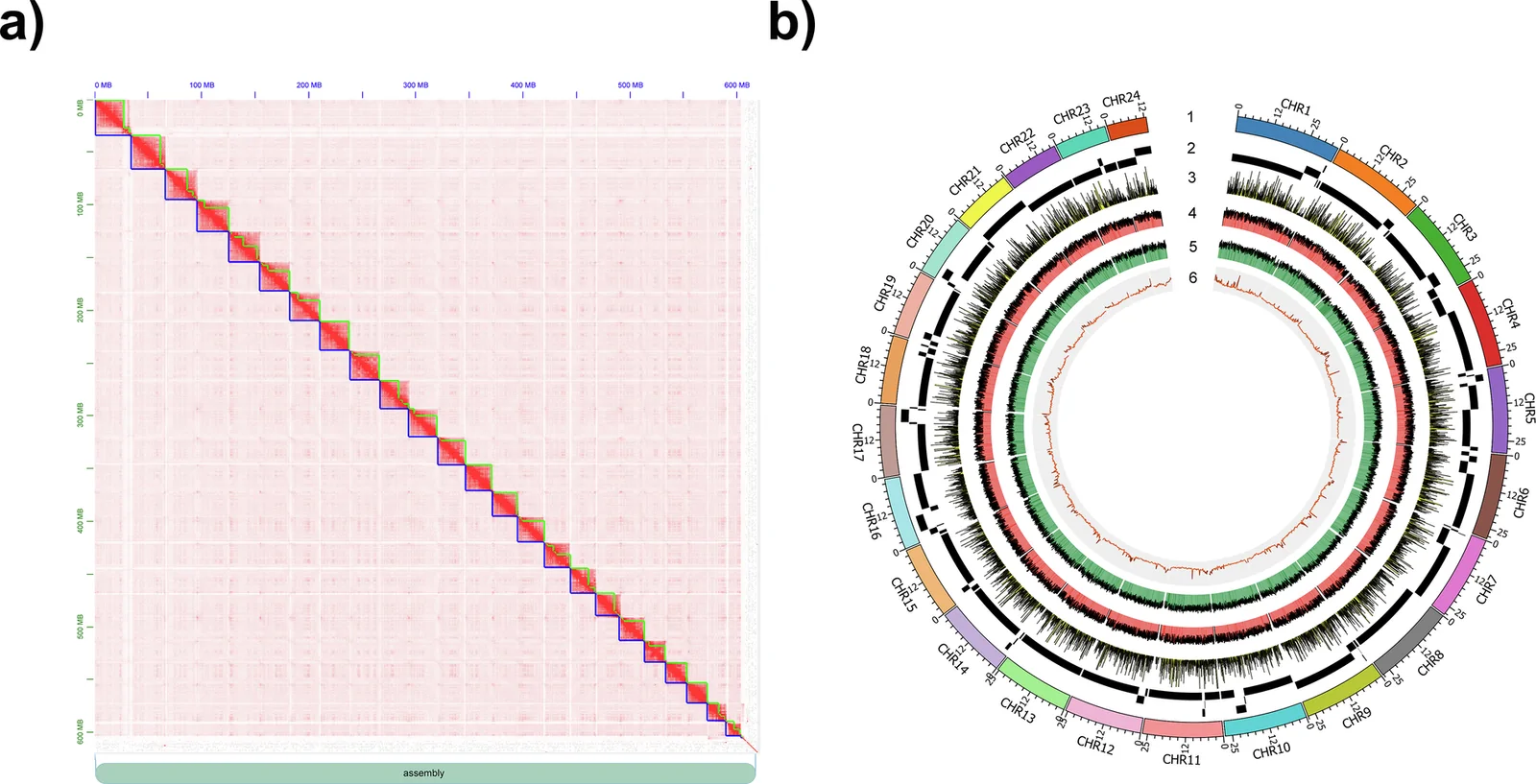
Hi-C contact matrix representing the assembled genome of *C. heberi* in 24 chromosome level scaffolds. The blue lines mark the scaffold borders and the green line marks the contig borders. **(b)** Circos plot representing various attribute of *C. heberi* genome. From the outermost: Track 1: The 24 chromosomes of *C. heberi* genome. Track 2: Contigs corresponding to the 24 chromosomes represented as tiles. Track 3: The protein encoding genes shown as a histogram. Track 4: Full length isoform sequences supporting the genes of *C. heberi* shown as histogram. Track 5: Transcripts generated from RNAseq data supporting the genes of *C. heberi* shown as line diagram. Track 6: GC content of *C. heberi* genome shown as a line diagram plotted with 250 kb sliding window.

The Hifi reads from the three libraries were screened for adaptor and foreign DNA contamination using NCBI Foreign Contamination Screen tool^27^ (Table 5). The clean reads from the three libraries were combined to obtain about 135x coverage of Hifi reads. Hifiasm v0.19.9-r616 tool^28^ was used to assemble the clean reads to obtain a contig level assembly. The assembly was about 618.76 Mb long, comprising of 169 contigs and an N50 Value of 19.81 Mb. The contig assembly was again screened for removal of contaminating scaffold sequences using NCBI Foreign contamination screen tool^27^. The Hi-C reads were pre-processed using fastp v0.12.4 tool^21^ for removal of adaptor and quality trimming before utilising them for scaffolding of the contig level assembly. The scaffolding was performed using the YaHS v1.1 tool^29^ and Arima Genomics’ mapping pipeline. The final primary assembly consisted of 159 scaffolds representing the nuclear genome, summing upto 618.7 Mb with an N50 value of 26.72 Mb **(**Table 6**)** and one scaffold representing mitochondrial genome. The top 24 scaffolds of the *C. heberi* genome assembly covered 97.50% of the assembled genome, indicating a high-quality chromosome-level assembly (Fig. 4). Comparative cytogenetic patterns in Carangidae fishes have revealed that all species have diploid chromosome number 2n = 48^16^.

**Table 6:** Genome assembly statistics of ***C. heberi***.

	Contigs	Scaffolds
**Number**	169	159
**Total Length, bp**	618,760,295	618,716,925
**Longest sequence, bp**	28,308,433	34,751,465
**N50, bp**	19,808,942	26,721,333
**L50**	14	11
**GC, %**	43.23%	43.23%
**N’s per Kb**	0	0.0098

### Repeat prediction and masking

Repeat prediction and masking was performed using Repeat Modeler v2.0.5^30^ and Repeat masker v4.1.5^31^ as described earlier^8,10^. The assembled genome of *C. heberi* was subjected to *de novo* repeat prediction using Repeat Modeler v2.0.5^30^, which utilizes RECON, TRF^32^, and LTR Retriever tools to identify the repeat elements. A total of 1,792 repeat families identified by Repeat Modeler, were used to mask the repeat elements in the genome by Repeat masker v4.1.5^31^. The repeat regions covered 20.94% of the genome. The majority of the repeat elements were categorized as unclassified (13.59%), followed by retroelements (2.56%) (Table 7). The gene prediction was performed on the repeat masked genome excluding simple sequence repeats and low complexity repeats.

**Table 7:** Repeat profile of *C. heberi*.

**Sequences**	159		
**Total length**	618,716,925 bp		
**GC level**	43.23%		
**Bases masked**	129,542,057 bp (20.94%)		
**Repeat type**	**Number of elements**	**Length occupied, bp**	**Percentage of sequence, %**
**Retroelements**	41,268	15,809,850	2.56
**SINEs:**	462	218,248	0.04
**LINEs:**	24,609	9,859,271	1.59
L2/CR1/Rex	17,499	6,990,065	1.13
R1/LOA/Jockey	1,122	63,044	0.01
R2/R4/NeSL	264	100,835	0.02
RTE/Bov-B	2,270	1,144,889	0.19
L1/CIN4	832	355,672	0.06
**LTR elements:**	16,197	5,732,331	0.93
BEL/Pao	2,395	878,383	0.14
Ty1/Copia	41	47,700	0.01
Gypsy/DIRS1	3,529	1,637,682	0.26
Retroviral	6,273	1,927,958	0.31
**DNA transposons**	35,940	9,949,547	1.61
hobo-Activator	4,949	1,023,156	0.17
Tc1-IS630-Pogo	20,105	3,631,011	0.59
PiggyBac	1,672	119,143	0.02
Tourist/Harbinger	2,090	572,939	0.09
Other (Mirage, P-element, Transib)	27	11,418	0.00
**Rolling-circles**	1,138	425,652	0.07
**Unclassified:**	360,983	84,057,164	13.59
**Total interspersed repeats:**		109,816,561	17.75
**Small RNA:**	363	47,351	0.01
**Simple repeats:**	439,359	16,902,337	2.73
**Low complexity:**	44,942	2,350,156	0.38

### Gene prediction and annotation

The gene prediction pipeline included evidence from *ab initio* predictions, IsoSeq data, and the RNAseq data as described earlier^6,7^ with minor changes. The *ab initio* prediction was performed by Augustus v3.4.0 tool^33^. In the next step, the FLNC transcripts obtained from IsoSeq long read data was mapped to the repeat masked genome using GMAP v2021-12-17^34^ to generate hints file. The third step for the RNA-Seq reads, the quality trimmed reads were mapped to the repeat masked genome using Hisat v2.2.1^35^ and subsequently assembled to transcripts using Stringtie v2.2.1^36^. The transcripts were further processed with TransDecoder v5.7.0 to identify candidate coding regions as evidence for final gene prediction step. The hints generated from *ab initio*, RNA-seq alignments and IsoSeq alignments were combined using a weighted approach within Evidence Modeler v2.0.0^37^. A total of 30,354 protein encoding genes were predicted from this approach **(**Table 8**)** followed by functional annotation of the predicted genes. A homology-based approach against the Actinopterygii (Taxonomy ID: 7898) cohort of the NCBI’s non-redundant database using blastx tool^38^ was performed at first. This was followed by a protein domain and functional motifs-based annotation was performed using the InterProScan module of the OmicsBox tool^39^. Simultaneously, Egg-NOG mapper module^40^ of the OmicsBox tool^39^ was used for the an orthology based annotation. The annotations obtained by all the three approaches were merged to obtain functional annotations based on gene ontology (Table 9). The annotated sequences were further subjected to pathway identification based on KEGG pathway database^41^, which resulted in the identification of 10,834 PEGs linked to 342 pathways^42^. The completeness of the predicted genes was assessed using BUSCO v5.8.3 tool^43^ against Actinopterygii_odb12(2025-04-11) ortholog database with transcriptome mode. BUSCO analysis revealed completeness of 97.9% consisting of 94.8% (6834) of complete orthologs and 3.1% (224) of fragmented orthologs, and 2.1% (149) missing orthologs.

**Table 8:** The protein coding genes predicted in the genome.

Property	Value
Total sequence length	618,716,925
Number of genes	30,354
Number of exons	250,203
Number of introns	219,849
Number of CDS	30,354
Total gene length	280,073,599
Total exon length	44,045,556
Total intron length	236,467,741
Total CDS length	44,045,556
Shortest gene	150
Longest gene	99,745
Longest CDS	59,118
mean gene length	9,227
mean exon length	176
mean intron length	1,076
mean CDS length	1,451
% of genome covered by genes	45
% of genome covered by CDS	7
mean exons per mRNA	8
mean introns per mRNA	7

**Table 9:** Annotation statistics of predicted protein encoding genes.

Attributes	No. of Transcripts	Percentage
**Predicted Genes**	30,354	100.00
**Genes with InterProScan Hits**	23,808	78.43
**Genes with Blast Hits**	26,031	85.76
**Genes with EggNOG annotation**	14,267	47.00
**Genes with GO Annotation**	21,968	72.37
**Genes with GO Mapping**	11,493	37.86
**Genes with Enzyme code**	6,776	22.32
**Annotated genes (overall)**	26,497	87.29

### Isoform classification

**Fig. 5:**
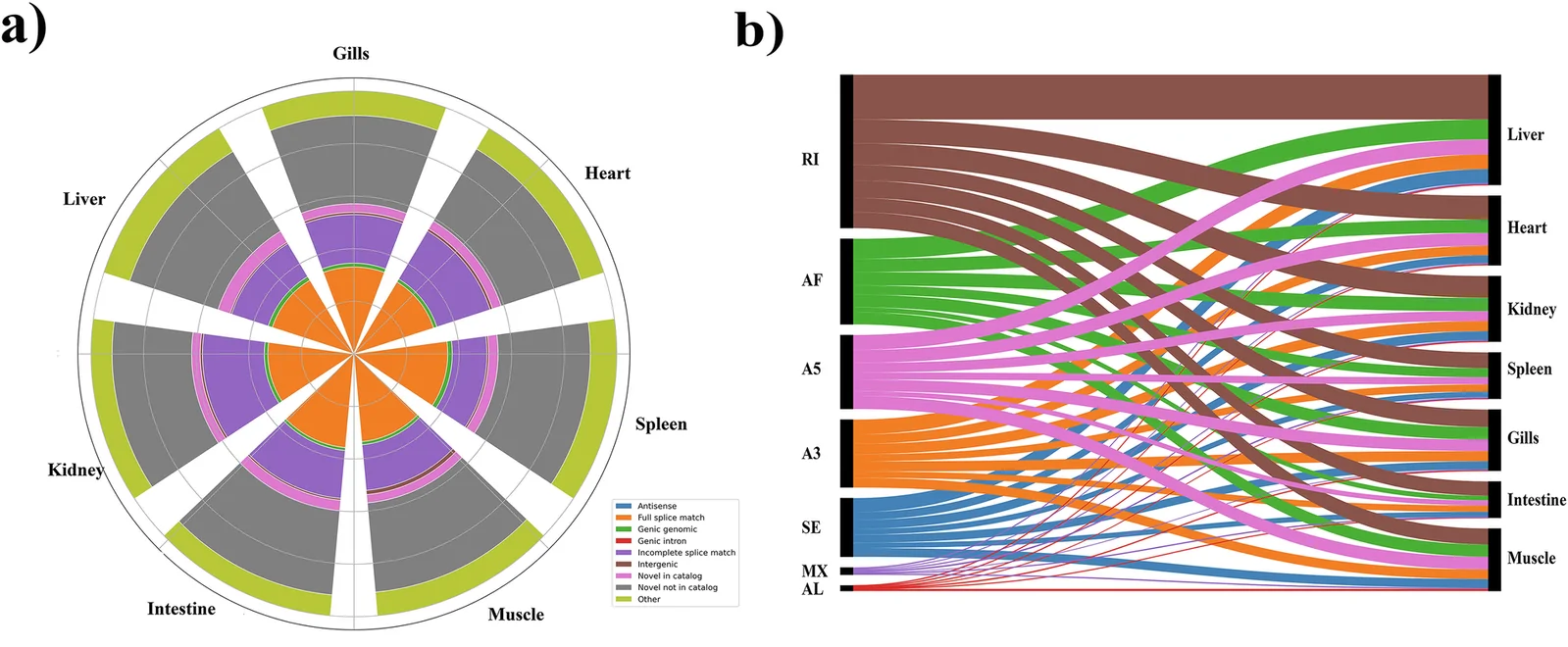
Tissue-specific isoform distribution and alternative splicing patterns in *C. heberi* revealed through Iso-Seq analysis. **a)** A stacked Rose chart depicting the classification of isoforms in *C. heberi* across various tissues in a stacked chart. The relative distribution of transcript isoforms is represented by stacked coloured bands, with each sector indicating a different tissue type (Gills, Heart, Spleen, Muscle, Intestine, Kidney and Liver). **b)** Map showing the alternative splicing events linked to different tissues of *C. heberi*. The right panel shows the tissue types and the left panel indicates the alternative splicing events. The width of the ribbon shows the absolute count abundance between each tissue and alternative splicing events.

The IsoSeq data was used to characterize the transcript diversity and identify isoform structures based on Isoseq analysis workflow containing three major steps comprising of read alignment, isoform collapsing and transcript classification. The FLNC reads were mapped to the assembled genome of *C. heberi* using a minimap2 wrapper for PacBio SMRT data, pbmm2 v1.17.0 (https://github.com/PacificBiosciences/pbmm2), with the ISOSEQ and sort flags enabled. IsoSeq v4.3.0^44^, which collapses redundant isoforms while preserving biologically important changes in transcript architecture, was then used to process the mapped bam file. In order to preserve transcript variation in the 5’ UTRs, which can indicate different promoter usage, the–do-not-collapse-extra-5exons option was employed. Transcript classification was then performed by using Pigeon: IsoSeq Transcript Classification Toolkit v1.1.0 (https://isoseq.how/classification/pigeon). The *prepare* module was first used to sort and index genomic features and transcripts.This step ensures that all records for each chromosome or scaffold are grouped together contiguously and create the necessary index files for the subsequent classification stage. Next the *classify* module was used to perform isoform classification, splice junction characterisations, and generate aggregated summary statistics by comparing novel transcript structures to reference annotations. The previously obtained FLNC counts were merged to improve the biological relevance of categorisation and raise the accuracy of isoform quantification prior to filtering step. Finally, the *filter* module was used to generate the high confidence classification and junction files. The resulting isoforms were classified into Antisense, Full splice match, Genic genomic, Genic intron, Incomplete splice match, Intergenic, Novel in catalogue, Novel not in catalogue, and other (Fig. 5a).

### Alternate splicing analysis

To further understand the transcriptome complexity and characterize the alternate splicing (AS) patterns, we analysed the AS events from the IsoSeq based isoform classification. The GFF3-formatted, filtered and collapsed isoform classification file obtained in the previous step was analysed using *generateEvents* module in SUPPA2 v2.3^45,46^, a transcript isoform quantification tool, for categorising the AS events. The major classes of AS events included Skipped Exon (SE), Alternative 5’/3’ Splice Site (A3 and A5, respectively), Mutually Exclusive Exons (MX), Retained Intron (RI), and Alternative First/Last Exon (AF and AL) (Fig. 5b). The splicing events across different tissues will reveal insights into the regulatory diversity and complexity of the *C. heberi* transcriptome.

## Data Records

The raw data generated in the current study including the Illumina DNA Short DNA reads (SRA Accession: SRR35799090), the Illumina RNASeq reads (SRA Accessions: SRR35799091 - 95, SRR35799097 and SRR35799098), the Hi-C Data (SRA Accessions: SRR35799088, SRR35799089 and SRR35799096), the PacBio Hifi reads (SRA Accessions: SRR35839761, SRR35839762 and SRR35839767) and the PacBio long read IsoSeq data (SRA Accessions: SRR35839763 – 66, SRR35839768 -70) were all deposited at NCBI SRA database under the Bioproject Accession PRJNA1338799^47^ with the SRA study accession number SRP632902^48^. The assembled genome was submitted under the genome category of NCBI with GenBank accession number GCA_054643105.1^49^. The contig assembly, genome assembly, mitochondrial genome sequence, predicted genes and annotations are uploaded to the Figshare repository^42^ 10.6084/m9.figshare.30636803.v2.

## Technical Validation

The contiguity and quality of the assembly was assessed based on the following parameters. (1) the N50 and L50 values at both contig and scaffold level, (2) the presence of full length mitochondrial genome in a single scaffold, (3) the assembly percentage in the longest scaffolds equivalent to the chromosome number of *C. heberi, (*4) the genome completeness assessed by BUSCO v5.8.3 tool^43^, (5) the quality value (QV) and error rate of the assembly assessed by Merqury v1.3 tool^50^, (6) the presence of telomeric repeats, (7) the genomic and transcriptomic raw reads representation stats, and (8) synteny with closely related species.

**Fig. 6:**
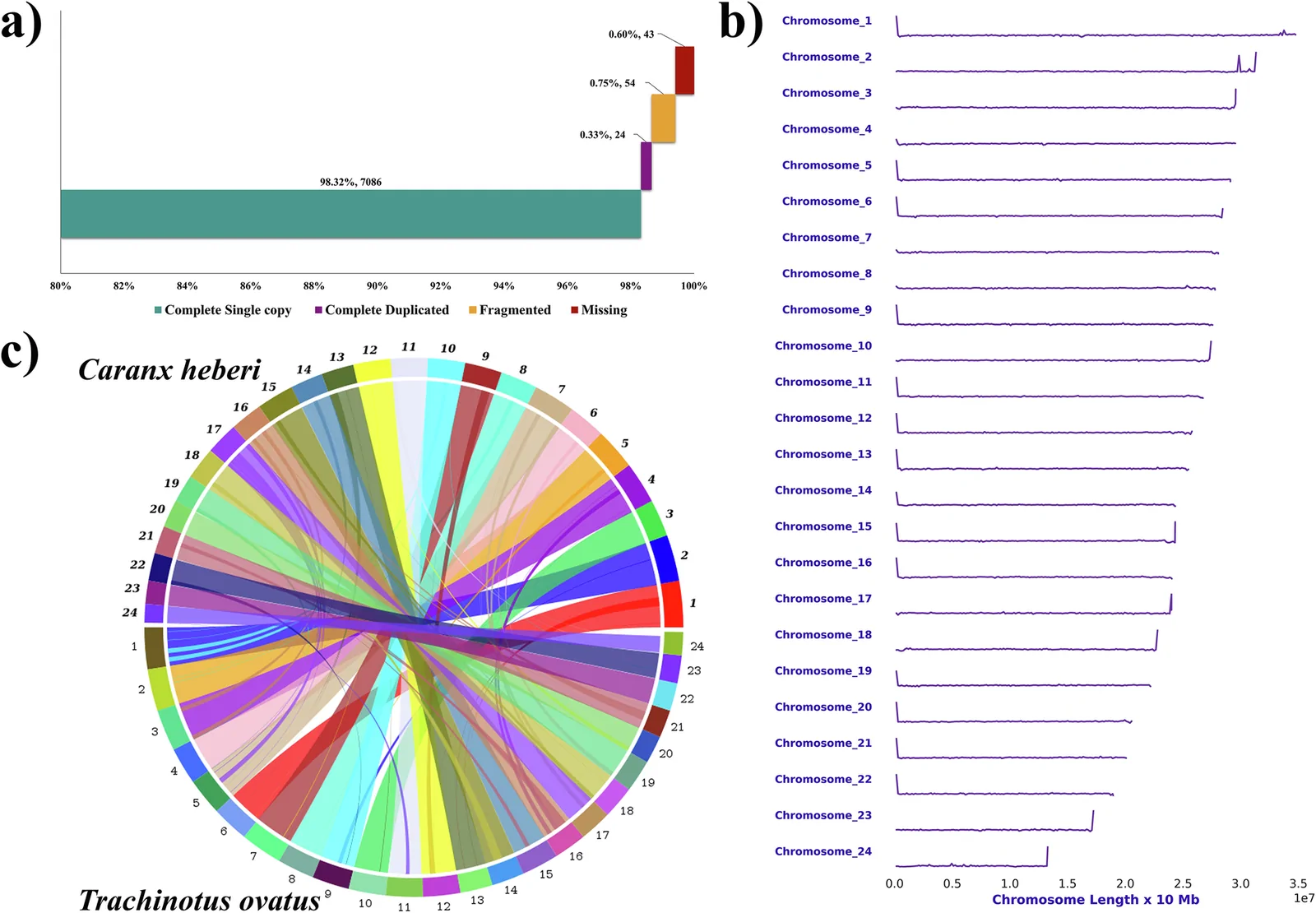
Genome assembly assessment and comparative genomic analysis of the chromosome-level genome of *C. heberi*. (**a**) Bar chart depicting the results of Genome completeness based on BUSCO analysis of *C. heberi* genome against Actinopterygii_odb12 orthologous database. (**b**) The graph representing the telomeric ends identified on the assembled chromosome level scaffolds of *C. heberi* genome. (**C**) Synteny plot between the 24 chromosome level scaffolds of two of the Carangoid fishes *C. heberi* and *Trachinotus ovatus*.

The contig level assembly consisted of 169 contigs with N50 value of 19.81 Mb and L50 of 14, and the scaffold level assembly consisted of 159 scaffolds with N50 of 26.72 Mb and L50 of 11 indicating a highly contiguous assembly at the contig level itself. The full-length mitochondrial genome of 16,591 bp, was also obtained from a single scaffold. The first 24 scaffolds encompassed 97.50% of the total assembly length indicating a chromosome level assembly. The genome completeness assessed using the BUSCO v5.8.3 tool^43^ with Actinopterygii_odb12(2025-04-11) ortholog database showed a genome completeness of 99.40% consisting of 98.7% (7110) of complete orthologs and 0.7% (54) of fragmented orthologs, and 0.6% (43) missing orthologs (Fig. 6a). Merqury v1.3 tool was used to assess the QV and error rate of the assembly. The QV was found to be 64.66 and an error rate of 3.42e-7, implying that there is 1 error base in 2.9 × 10^6^ bases indicating that the assembly is highly accurate. The telomere identification using tidk v0.2.0 tool identified telomeric ends of the scaffolds (Fig. 6b). The assembly was also validated by mapping the quality trimmed RNA-seq and DNA short reads to the assembled genome **(**Table 10**)**. Synteny analysis was performed using Symap v5.3.5 for *C. heberi* genome against *Trachinotus ovatus*. The 24 chromosomes of *C. heberi* consisting of 603,260,218 bp were mapped to 24 chromosomes of *T. ovatus* consisting of 651,835,818 bp (Fig. 6c). A total of 101 syntenic blocks were generated with 24 blocks > 10 Mb, 74 ranging between 100 kb and 10 Mb and 3 < 100 kb. Coverage statistics indicated that 97% of the *C. heberi* genome sequence was covered by the syntenic blocks corresponding to 98% of *T. ovatus* genome, reflecting a high genome wide correlation between the two species.

**Table 10:** Read alignment of Illumina short read DNA and RNA datasets generated in this study against the *C. heberi* genome.

S. No.	Data	Alignment rate (%)	SRA Accession numbers
**1**	DNA- Illumina Short reads	99.95	SRR35799090
**2**	RNA - Muscle	99.90	SRR35799098
**3**	RNA - Kidney	99.99	SRR35799097
**4**	RNA - Intestine	99.97	SRR35799095
**5**	RNA - Liver	99.97	SRR35799094
**6**	RNA - Gills	96.72	SRR35799093
**7**	RNA - Heart	99.90	SRR35799092
**8**	RNA - Spleen	99.81	SRR35799091

### Ethics statement

The animal study was reviewed and approved by the Institute Animal Ethics Committee (CIBA/IAEC/2023-1 dated 15.02.2023) of ICAR—Central Institute of Brackishwater Aquaculture, Chennai, India.

## Data Availability

The raw datasets generated were submitted under the SRA study SRP632902 and Bioproject Accession PRJNA1338799. The assembled genome was submitted under the genome category of NCBI with GenBank accession number GCA_054643105. The contig assembly, genome assembly, mitochondrial genome sequence, predicted genes and annotations are uploaded to the Figshare repository, 10.6084/m9.figshare.30636803.v2.

## Code availability

All data processing commands and pipelines were carried out per the instructions and guidelines provided by the relevant bioinformatics software. No custom scripts or code were utilised in this study.
